# Distinct Gene Expression Patterns of Ion Channels and Cytokines in Rat Primary Sensory Neurons During Development of Bone Cancer and Cancer Pain

**DOI:** 10.3389/fnmol.2021.665085

**Published:** 2021-05-06

**Authors:** Mingzhu Zhai, Shaomin Yang, Simin Lin, Hanxu Zhu, Lihong Xu, Huabao Liao, Xue-Jun Song

**Affiliations:** ^1^School of Life Science and Technology, Harbin Institute of Technology, Harbin, China; ^2^Key Laboratory of Carcinogenesis and Translational Research (Ministry of Education of China), Peking University Cancer Hospital and Institute, Beijing, China; ^3^SUSTech Center for Pain Medicine, School of Medicine, Southern University of Science and Technology, Shenzhen, China; ^4^Department of Perioperative Medicine, SUSTech Hospital, Southern University of Science and Technology, Shenzhen, China; ^5^Department of Pain Medicine, Shenzhen Nanshan Hospital, Huazhong University of Science and Technology, Shenzhen, China; ^6^Department of Laboratory Animal Center, Southern University of Science and Technology, Shenzhen, China

**Keywords:** cancer pain, dorsal root ganglion, RNA-sequence, cytokines, ion channels, neuropathic pain, hyperalgesia, allodynia

## Abstract

Cancer and cancer pain processes a major clinical challenge and the underlined mechanisms of pathogenesis remain elusive. We examined the specific changes in the transcriptomic profiles in the dorsal root ganglion (DRG) neurons of rats with bone cancer and bone cancer pain (BCP) using RNA sequencing technology. The bone cancer and BCP was induced by tumor cells implantation (TCI) into the tibia bone cavity in adult female rats. One week after treatment, TCI caused up- and down-regulation of thousands of genes in DRG. These genes were mainly involved in the immune process, inflammatory response, and intracellular signaling transduction of carbohydrate and cytokine. The cAMP and calcium signaling pathways were the major processes in the initial responses. Differentially expressed gene (DEG) analysis further showed that the genes for ion channels increased during day 1-7, while the genes for cytokine signaling pathways sustainedly increased during day 7-14 after TCI. The time courses of gene expression for ion channels and cytokines support their distinct roles in the early induction and late maintenance of BCP development. In addition, among the top 500 up- and down-regulated genes, 80-90% were unique for bone cancer pain as well as neuropathic and inflammatory pain, while less than 2% were shared among the three different forms of pain. This study reveals the uniqueness of mechanisms underlying bone cancer with pain, which is, to a large extent, differently from pain after acute inflammatory and nerve injury and provides novel potential targets of DEGs for bone cancer with pain.

## Introduction

Bone cancer and bone cancer pain (BCP) continues to be a major clinical challenge. Despite decades of thorough study and continuous efforts, the underlined cellular and molecular mechanisms remain elusive and the clinical approaches for treating bone cancer and BCP are limited (van den Beuken-van Everdingen et al., 2007; Breivik et al., 2009). BCP happens in patients with primary bone sarcomas and predominantly occurs with distant metastases of non-bone cancer, including lung, breast, or prostate cancer (Kane et al., 2015). The incidence of BCP is estimated at around one-third of the patients with bone metastases (van den Beuken-van Everdingen et al., 2007; Scarborough and Smith, 2018). It is pivotal and urgent to elucidate the pathogenesis of BCP and identify candidates of therapeutic targets for translational medicine. Mechanisms of BCP are complex and have been considered involving a combination of inflammatory and neuropathic factors, as well as imbalance in bone metabolism, specifically (Falk and Dickenson, 2014). Cancer cells act to cause BCP in many ways. For instance, cancer cells and their associated stromal cells can release a wide variety of biochemical factors, which in turn initiate activation of nociceptors (Julius and Basbaum, 2001; Mantyh, 2006; Joyce and Pollard, 2009). Cancer cells invade into tissue and induce nerve injury, indicating a neuropathic origination for cancer pain (Peters et al., 2005). Tumor-induced bone destruction and acidic microenvironment by osteolysis result in sensory neuron hyperexcitation by activating acid-sensing nociceptors. The biochemical and microenvironmental changes further elicit sensitization and reconstruction of peripheral nerve endings and dorsal root ganglion (DRG) neurons that eventually result in pain.

DRG neurons, as the first-order neurons in the somatosensory afferent pathway, transduce and integrate diverse stimuli and signals from the peripheral to central nervous system. Emerging evidence indicates that alterations in gene expression including ion channels (Qiu et al., 2012, 2014; Wu et al., 2012) and other signaling pathways in DRG (Xu et al., 2013; Zhu et al., 2014; Fang et al., 2015; Guan et al., 2015; Wei et al., 2017; Huang, 2018; Liu et al., 2018a) contribute to the induction and maintenance of BCP. For instance, upregulation of P2X3 (Wu et al., 2012), TRPV1 (Han et al., 2012), Nav1.8 and Nav1.9 (Qiu et al., 2012), and ASIC3 (Qiu et al., 2014) are reported to be critical important in BCP development. Recent studies show that activation of cytokines including IL-6, IL-18, and TGFβ (Xu et al., 2013; Zhou et al., 2016; Liu et al., 2018a), and cAMP-PKA (Zhu et al., 2014), PI3K/Akt (Guan et al., 2015), BDNF (Huang, 2018), Wnt (Zhang et al., 2013), and Hedgehog (Liu et al., 2018b) signaling pathways play important roles in the peripheral mechanism of BCP. However, an overall global gene-level alteration of ion channels and cytokine signaling pathways in DRG that may contribute to bone cancer associated BCP and act as potential therapeutic targets has not been well examined and remains elusive. By means of RNA-sequence techniques, we have identified differentially expressed genes (DEGs) of ion channels and cytokine signaling pathways in rat DRG during the development of bone cancer and BCP produced by tumor-cell implantation (TCI) into the tibia bone cavity. The genes for ion channels transiently altered in the initial early phase after TCI treatment. In contrast, genes for cytokine signaling pathways sustainedly increased from the initial to the later phases of BCP after TCI and were mainly involved in the immune process and inflammatory response as indicated by GO and KEGG analysis. Our study demonstrates distinct roles of the ion channels and cytokines in the development of bone cancer and induction and maintenance of BCP and presents a global description of transcription profile changes in DRG and provides a new insight into the underlying mechanisms of bone cancer with pain and guidance of rational clinical medication.

## Materials and Methods

### Ethics Statement and Animals

All procedures were performed following the regulations of the ethics committee of the International Association for the Study of Pain and the Guide for the Care and Use of Laboratory Animals. The experiments were designed to minimize suffering and the number of animals used and approved by the Southern University of Science and Technology (SUSTech) Animal Care and Use Committee. Adult, female Sprague-Dawley rats (160-180 g-wt) purchased from Guangdong Province Animal Center were used in this study. Animals were then housed (2 per cage) at room temperature (20-24°C) and exposed to a 12-h light/dark cycle with ad libitum access to food and water in the University Animal Care Center.

### Model of Bone Cancer and BCP

**Bone cancer and** BCP was induced by tibia bone cavity tumor-cell implantation (TCI), which produced bone cancer-related thermal hyperalgesia, mechanical allodynia, spontaneous and movement-evoked pain behaviors, and bone destruction. Tumor cells were extracted from the ascitic fluid of rats that had previously received Walker-256 mammary gland carcinoma cells. The tumor cells (1 × 10^5^ cells/ μL, 5 μL) were injected into the intramedullary space of the left tibia to induce bone cancer pain in rats. The protocol of this bone cancer and BCP model was similar to that previously described (Medhurst et al., 2002; Liu et al., 2011). In brief, rats were anesthetized with sodium pentobarbital (50 mg/kg intraperitoneally), and an approximately 1-cm long superficial incision was made near the knee to expose the lateral tibia. A 23-gauge needle, which was connected with a 25-μL microinjection syringe containing a tumor cell sandwich with 5 μL air, 5 μL suspension of tumor cells (5 × 10^5^), and 5 μL air, was inserted into the tibia cavity. The contents of the syringe were slowly injected into the tibia cavity, and the needle was kept for 2 min for cell diffusion. Then, the needle hole was sealed with bone wax while pulling out the needle. The bone surface of the injection site was sealed with dental bone cement to prevent the leakage of tumor cells. Boiled tumor cells were injected as the sham control. Experimenters who performed behavioral tests, preparation of DRG tissues for RNA-sequence were blinded to this surgery. Total of 24 animals received TCI treatment and 8 for control.

### Assessment of Bone Cancer-Related Pain Behaviors

Pain-like behaviors including spontaneous pain and those evoked by movement, mechanical and thermal stimulation were evaluated in rats with TCI and sham control treatment. Mechanical allodynia was indicated by a significant decrease in the threshold of paw withdrawal to mechanical indentation of the plantar surface of each hind paw. The withdrawal was measured by von Frey filaments (Aesthesio, Ugo, Italy) with a protocol similar to that described previously (Chaplan et al., 1994; Zhang et al., 2013). In brief, the animals were placed individually beneath an inverted ventilated cage with a metal-mesh floor. The von Frey filaments were applied perpendicularly to the mid-plantar surface of each hind paw from beneath until the paw was withdrawn. The duration of each stimulus was approximately 1-2 s and the interstimulus interval was approximately 10-15 seconds. Thermal hyperalgesia was determined by the significantly shortened latency of foot withdrawal in response to heat stimulation. An analgesia meter (Model 390G; IITC Life Science) was used to provide a heat source. The protocol was similar to that described previously (Zhang et al., 2013). In brief, each rat was individually beneath an inverted cage (22 cm × 15 cm) with a smooth, temperature-controlled glass floor. The heat source was focused on a portion of the hind paw, and a radiant thermal stimulus was delivered to that site. The stimulus shut off automatically when the hind paw moved (or after 25 s to prevent tissue damage). The intensity of the heat stimulus was maintained constant throughout all experiments. Thermal stimuli were delivered 4 times to each hind paw at intervals of 5-8 min. Spontaneous and movement-evoked pain-like behaviors were also analyzed (Zhang et al., 2013). Spontaneous-nocifensive behaviors were evaluated by measuring flinching over 2 minutes of observation. Movement-evoked pain was assessed by measuring the limb use during spontaneous ambulation, which was scored on a scale of 0 to 4: 0 = normal use; 1 = slightly limping; 2 = clearly limping; 3 = no use of the limbs (partial); and 4 = no use of the limbs.

### Micro-CT Analysis of Bone Metabolism of the Tibia

Micro-CT analysis was performed on tibias taken from TCI and sham rats on the 7 and 14 days, respectively, after TCI using a Bruker scanner (Skyscan 1276) with the 100-kVp source (Wang et al., 2019). Quantification of micro-CT data was calculated for the proximal tibia of rats with TCI or sham treatment. Parameters including BV/TV, Tb.Th, Tb.Sp, Tb.N, and BMD were analyzed within a region 2-10 mm, and parameter Ct.Th within a region 16-18 mm proximal to the growth plate, respectively.

### Total RNA Extraction

Under deep anesthesia, L3-L5 DRGs ipsilateral to TCI or sham treatment animal were dissected out and pooled as one sample. DRG tissues were taken at different time points, i.e., the postoperative/tumor-cell injection 1, 7, and 14 days. Total RNA was isolated with Trizol reagent (Sigma-Aldrich, St. Louis, MO, USA) according to the manufacturer’s instructions and followed by chloroform extraction and isopropanol precipitation. The extracted RNA was about 2 μg and stored at −80°C deep freezer until use.

### RNA Sequence and Data Analysis

The preparation of the cDNA library from each sample and the sequencing was performed by the Beijing Genomics Institute (BGI, Shenzhen, China). The cDNA originating from the RNA fragments were paired and sequenced using the high throughput sequencing platform of BGISEQ-500 and ∼33 M raw reads per sample were obtained on average. The sequencing reads containing low-quality, adaptor-polluted, and high content of unknown base (N) reads were removed. Clean reads were then mapped to reference using HISAT/Bowtie2 tool (Langmead et al., 2009). Genes expression level was quantified by a software package called RSEM (Dewey and Li, 2011). Based on the gene expression level, we identified the differentially expressed genes (DEGs) between groups using DEGseq2 algorithms. Gene ontology (GO) analysis and Significantly enriched Kyoto Encyclopedia of Genes and Genomes (KEGG) pathways of gene functional annotation clustering was performed by ClusterProfiler (Yu et al., 2012) using a modified Fisher’s exact test followed by Benjamini-Hochberg multiple hypothesis testing correction and rattus norvegicus as background, default options, and annotation categories. The sequencing results have been submitted to the Gene Expression Omnibus (GEO) database of NCBI, and the assigned accession number is GSE149648.

### Statistical Analysis

All data were represented as means ± SEM. Statistical analysis was performed with two-way repeated measure ANOVA followed by Sidak’s multiple comparisons test or Student’s *t*-tests using Prism 7 software. Two-tailed *P* values less than 0.05 were considered to be significantly different.

## Results

### Bony Destruction and Painful Behavioral Manifestations Following TCI Treatment

**FIGURE 1:**
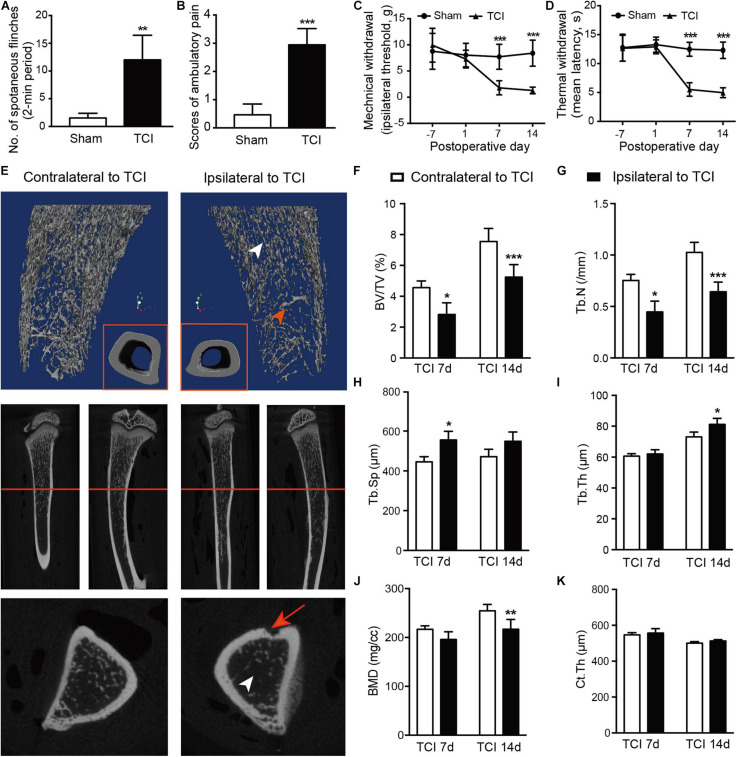
TCI treatment-induced painful behaviors and changes in morphology and density of tibia. **(A,B)** Spontaneous painful behaviors manifested as flinching and movement evoked pain manifested as reduced limb use. Averaged measurements of day 7 and 14. Eight rats included in each group. Student’s *t*-tests, ** *P* < 0.01, ***P < 0.001 vs. sham group. **(C,D)** Mechanical **(C)** and thermal **(D)** hypersensitivity manifested as lowered mechanical threshold and shortened latency of thermal withdrawal. Eight rats included in each group. Two-way repeated measure ANOVA, ***P < 0.001 vs. sham group. **(E)** Morphological changes presented by micro-CT images of 3-dimension reconstruction graph (blue background) and 2-dimension photos (black background) by coronal and sagittal scanning of the ipsilateral and contralateral tibia in TCI rats on day 14. Arrowheads: white, the zone of less dense bone; red, newly formed bone tissue; red arrow: site of cancer cell injection; red line: cross-section. **(F-K)** Statistical analysis of bone parameters measured by microCT on day 7 (*n* = 3) and 14 (*n* = 6), separately. Student’s *t*-tests between the two groups measured on the same day, * *P* < 0.05, ** *P* < 0.01, ***P < 0.001 ipsilateral vs. contralateral to TCI. BV/TV = bone volume/total volume, bone volume fraction **(F)**. Tb.N = trabecular number **(G)**. Tb.Sp = trabecular separation, the thickness of cavities **(H)**. Tb.Th = trabecular thickness, the average thickness of all bone voxels **(I)**. BMD = Bone mineral density **(J)**. Ct.Th = Cortical thickness, the average thickness of cortical bone **(K)**.

We started by examining and confirming alterations of bone density and the associated painful syndromes in rats by TCI. As expected, TCI treatment produced painful behaviors in all the TCI-treated animals manifested as spontaneous and evoked pain. Spontaneous pain was evidenced by the significantly increased spontaneous flinching (Figure 1A) and reduced limb use (Figure 1B) measured on day 7 and 14 after TCI. The evoked-pain was evidenced by significantly increased responses to the mechanical and thermal stimulation, respectively, as so-called mechanical allodynia (Figure 1C) and thermal hyperalgesia (Figure 1D). We further examined bone metabolism of the tibia by micro-CT (Figures 1E–K). MicroCT imaging analyses showed that TCI significantly reduced the trabecular bone (Figure 1E). Both the bone volume fraction BV/TV (Figure 1F) and trabecular number (Tb.N; Figure 1G) were significantly reduced on day 7 and 14 after TCI treatment. The trabecular separation (Tb.Sp; Figure 1H) and trabecular thickness (Tb.Th; Figure 1I) was increased, the bone mineral density (BMD) was decreased (Figure 1J), while the difference between cohorts for cortical thickness was not changed (Ct.Th; Figure 1K). These results show clear behavioral signs of BCP and bony destruction following TCI treatment.

### An Overview of DEGs Profiles in DRG

**FIGURE 2:**
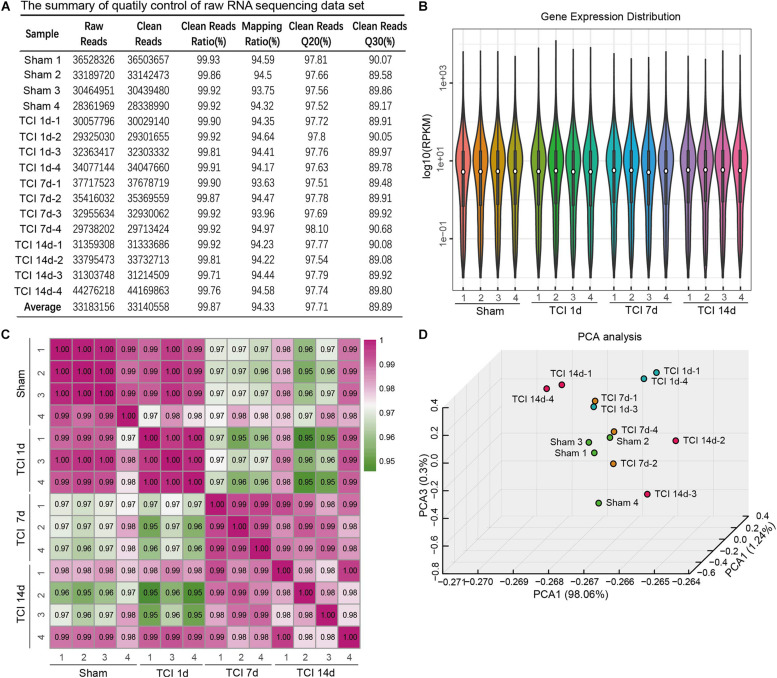
The quality of RNA-seq data from each of the DRG samples. **(A)** Summary of raw RNA sequencing data set from16 samples, including raw reads number, clean reads number, clean reads ratio, mapping ratio, and percentage of clean reads as well as Q20 (Phred quality scores Q) and Q30. **(B)** Distribution of gene expression levels in each of the samples. RPKM: Reads Per Kilobase Million. **(C)** Heatmap of the correlations between every two samples from each of the groups with the Pearson test. **(D)** Principal component analysis (PCA) of all the samples.

To evaluate the dynamic change of transcriptome profiles in DRG after TCI treatment, we performed RNA-Seq and gene analysis to detect gene expression patterns of the bone cancer and the associated pain-related ion channels and cytokines at different stages of bone cancer and BCP development. The transcriptome data was plotted at different time points, day 1, 7, and 14 after TCI, and sham control. L3-L5 DRGs from the ipsilateral side of an animal in the different groups were collected as one sample for the subsequent transcriptome analysis. The summary of quality control of raw RNA sequencing data set is shown in Figure 2A. On average, about 33,140,558 clean reads were collected and mapped to the reference genome using HISAT after filtering low-quality data and with a mapping rate of round 94∼95%. Comparison of the distribution of the gene expression level of each sample and the uniformity of the dispersion of gene distribution for each sample supports that the samples are comparable (Figure 2B). The correlation of gene expression level among samples has been used as a key criterion to test whether the experiments are reliable and whether the samples chosen reasonable. We calculated the correlation value between every two samples based on normalized expression results and draw a correlation heat map (Figure 2C). Sample 2 from Day 1 and sample 3 from Day 7 after TCI treatment were excluded from the final statistics due to low correlation (R < 0.8). Besides, the principal component analysis (PCA) was performed to assess the degree of data dispersion. DRG tissues collected from groups of sham control and those 1, 7, and 14 days after TCI treatment were dramatically separated from each other’s (Figure 2D).

**FIGURE 3:**
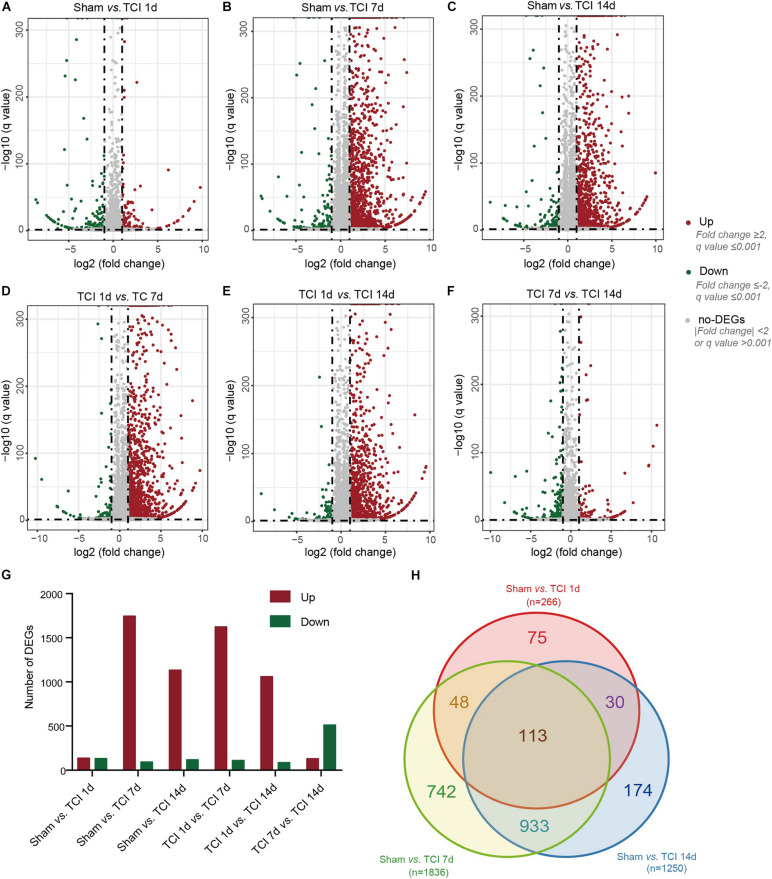
Dynamic alterations of DEGs in DRG ipsilateral to TCI treatment. **(A-F)** Volcano plot of all the differentially expressed genes from pairs of comparable groups. Log2 (fold change) is plotted as the abscissa and –log10 (Corrected *P* Value) is plotted as the ordinate. Each dot represents a single gene (red dots = up-regulated genes; green dots = down-regulated genes; gray dots = genes with no significant difference). **(G)** Comparison of up- and down-regulated DEGs between groups. **(H)** A Venn diagram presents DEGs that are unique or shared in each of the paired groups.

Next, we examined transcriptome changes in the different stages of bone cancer and BCP development. DEGs screening was performed to filter the DEGs with the cut-offs of | log2 FC| ≥ 1 and normalized *P* value, q value ≤ 0.001 between two comparable groups for further functional analysis. The representative distributions of up- and down-regulated or no-change genes between the compared groups were shown in the volcanos in Figures 3A–F, in which the red- and green-dots represent up- and down-regulated genes, respectively. One day after TCI treatment when there was no bone destruction and painful behavior exhibited, there were 136 genes up-regulated and 130 genes down-regulated. On day 7 after TCI when TCI-induced pain had been well developed (also see Figures 1A–D) with bone destruction (Figures 1E–K), differential gene expression reached its peak. There were 1744 genes up-regulated and 92 genes down-regulated compared to the sham group. These dramatic changes of bulk of genes lasted for at least one week, there were 1133 genes up-regulated and 117 genes down-regulated detected on day 14 after TCI compared to the sham group. We also noted that, compare to day 7 after TCI, there were 129 DEGs up-regulated and 511 DEGs down-regulated on day 14 (Figure 3G). These results indicate that a large number of genes in DRG were involved in the development of bone cancer and BCP. Interestingly, these gene expression alterations are highly synchronized with the development of BCP and shows different patterns among the different stages of BCP development, but not always synchronized with the development of bone cancer exhibited with bone destruction (Figure 3G). We also analyzed the unique or shared genes between each paired group, and the number of DEGs were represented by a Venn diagram (Figure 3H). On day 1, 7, and 14 after TCI, 75, 742, and 174 genes were found unique on each of these days, while 113 genes were shared in the three examined days. These results show majority of the altered genes, approximately 82% [817 (75+742) out of 991(817+174)], were involved in the early development of bone cancer and BCP after TCI treatment, while 174 of the altered genes were responsible for maintaining the late persistence of BCP and perhaps the cancer development. These findings reveal that the major gene expressional and functional changes occur in the first week after TCI, suggesting that the altered gene expression occurred in the early-stage after TCI and may play a decisive role in cancer development and BCP induction.

### Gene Ontology Analysis of DEGs

**FIGURE 4:**
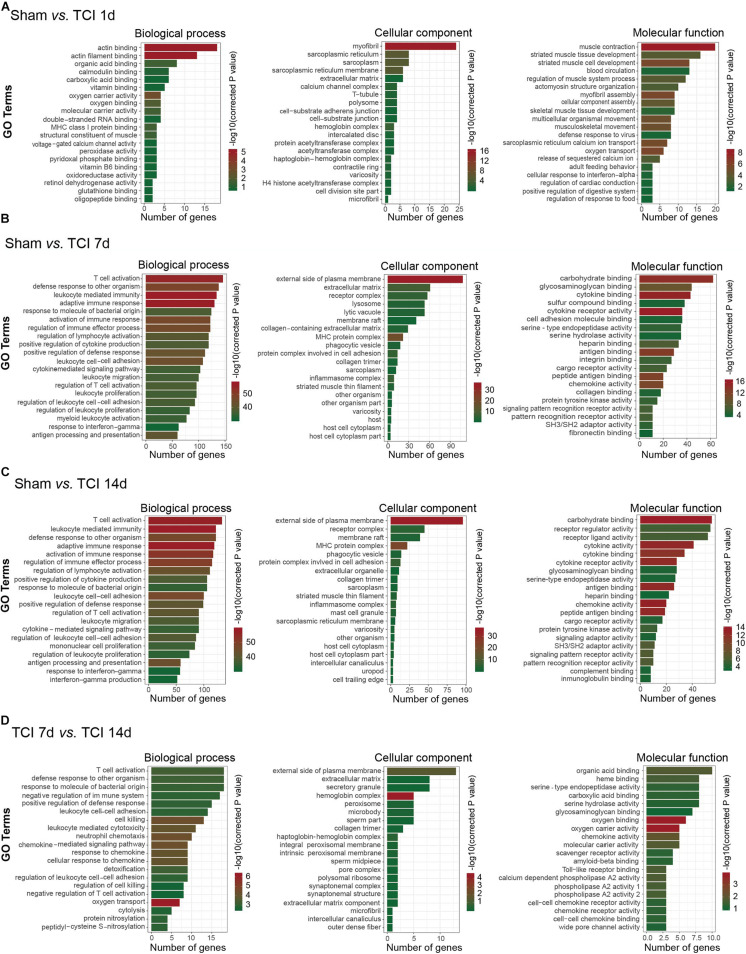
Functional analyses of DEGs in DRG from different stages. **(A–D)** Gene ontology (GO) analysis showing the enrichment of DEGs in biological process, cellular component, and molecular function from pairs of comparable groups. The top 20 significantly enriched GO terms determined by –log10 (corrected *P* value) are plotted as the ordinate and the enriched gene number is plotted as the abscissa.

**FIGURE 5:**
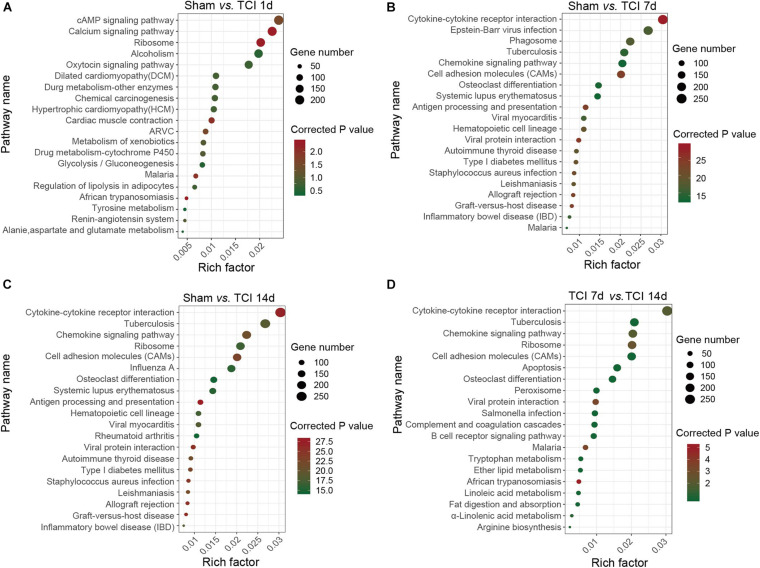
KEGG classifications of DEGs in DRG. **(A–D)** The comparison of pathway enrichment from pairs of comparable groups. The top 20 KEGG terms determined by the rich factor is plotted as the ordinate. The rich factor is plotted as the abscissa. The gene number involved in each pathway is represented by the size of the dots. ARVC: Arrhythmogenic right ventricular cardiomyopathy.

To further understand the biological process, cellular component, and molecular functions of the DEGs associated with the development of bone cancer and BCP following TCI treatment, we used GO analysis to perform enrichment analysis and classifications. GO analysis identified that biological processes including “actin binding” and “actin filament binding”, the cellular component “myofibril”, and the molecular function term “muscle contraction” were significantly enriched on day 1 after TCI (Figure 4A). These results suggest that these DEGs associated with muscle tissue regeneration responded to TCI treatment quickly and were involve in the initial responses to cancer development and the initiation of BCP induction. Further, on day 7 and 14 after TCI, the enriched biological processes were those representing immune processes, including “T cell activation”, “defense response to other organisms”, “leukocyte mediated immunity”, and “adaptive immune response”, the enriched cellular components were the “external side of plasma membrane”, and the molecular function terms were “carbohydrate binding”, “cytokine binding”, and “cytokine receptor activity” (Figures 5B–D). These results demonstrate strong immune processes and the intracellular signaling transduction of carbohydrate and cytokine starting from day 7 and lasted at least to the 14 days after TCI treatment that were critical period of time for the development of bone cancer and BCP. Token together, these findings suggest important mechanisms underlying the development of bone cancer and BCP induction and that different transcriptome profiles are involved in the initiation/induction within a couple of days and the subsequent development of BCP.

### Analysis of TOP20 KEGG Pathways

To identify the major signaling pathways involved in the initial responses of DRG to development of cancer and BCP, we continued to analyze DEGs for KEGG pathway enrichment. Our results showed that the significantly enriched DEGs were in the classifications of “cAMP signaling pathway”, “Calcium signaling pathway”, and “Ribosome” on day 1 after TCI (Figure 5A). However, on day 7 and 14 after TCI, the significantly enriched DEGs were “Cytokine-cytokine receptor interaction (CCRI)”, Epstein-Barr virus infection”, “Phagosome”, and “Tuberculosis” (Figures 5B–D). Notably, CCRI exhibited the most significant changes on days 7 and 14 after TCI. These results suggested that cAMP and calcium signaling pathways are the major processes in the initial responses of DRG to the TCI treatment including cancer development and initiation of BCP induction, while cytokine signaling and other cell-defense related pathways mainly contribute to the development of bone cancer and the subsequent development of BCP, in which CCRI may play the most important roles.

### Divergent Gene Expression Patterns of Ion Channels Following TCI Treatment

**FIGURE 6:**
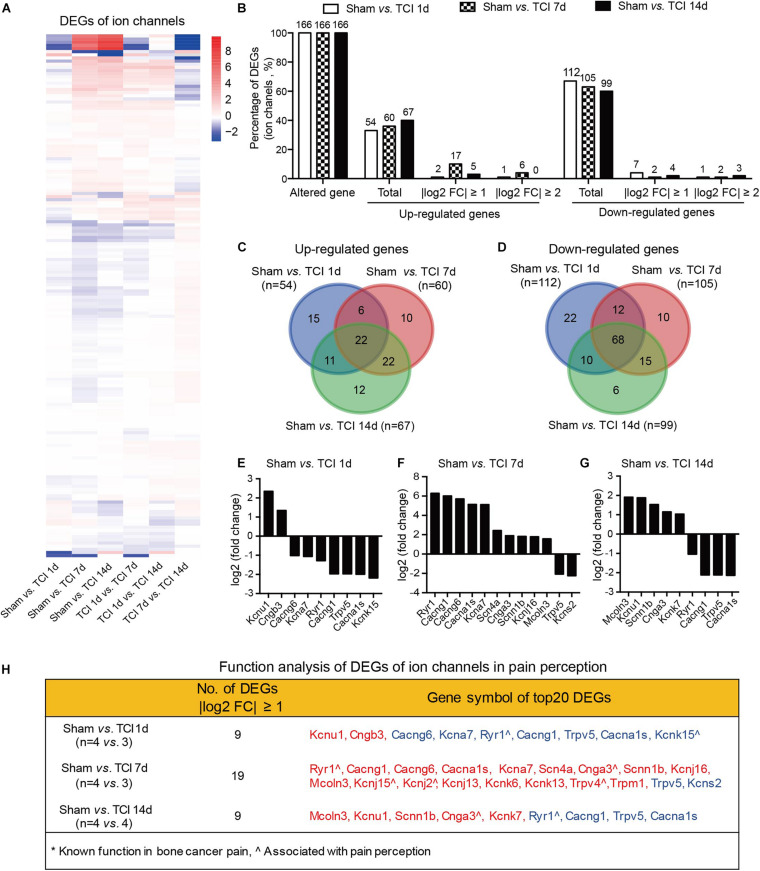
Dynamic gene expression profiles of ion channels during the development of BCP. **(A)** Heatmaps depicting expression patterns for genes encoding ion channels from pairs of comparable groups. Up- or down-regulated genes are presented as the indicated color bars (red to blue). **(B)** Histogram showing the statistics of DEGs for ion channels. The number and percentage of the altered gene, up- and down-regulated gene in total, with | log2 FC| ≥ 1 and | log2 FC| ≥ 2. **(C,D)** Venn diagrams shows numbers of up- **(C)** and down-regulated **(D)** DEGs that are unique or shared in each paired group. **(E-G)** Plot data showing the top 10 up- or down-regulated genes for ion channels, ranked by multiple of difference (only genes with | log2 FC| ≥ 1 were plotted). **(H)** Function analysis of top 20 DEGs for ion channels that are considered associated with pain perception. DEGs with different symbols from the encoded proteins include Kcnu1 (Kca5.1), Cngb3 (cyclic nucleotide-gated cation channel beta-3), Cacng6 (voltage-dependent calcium channel gamma-6 subunit), Kcna7 (Kv1.7), Cacng1 (voltage-dependent calcium channel gamma-1 subunit), Cacna1s (Cav1.1), Kcnk15 (potassium channel subfamily K member 15), Scn4a (Nav1.4), Cnga3 (cyclic nucleotide-gated cation channel alpha-3), Scnn1b (SCNEB), Kcnj16 (Kir5.1), Mcoln3 (TRPML3), Kcnj15 (Kir4.2), Kcnj2 (Kir2.1), Kcnj13 (Kir7.1), Kcnk6 (TWIK-1), Kcnk13 (THIK-1), Kcns2 (Kv9.2).

We know that certain ion channels have been proved to be involved in the development of BCP (Julius and Basbaum, 2001; Peters et al., 2005; Mantyh, 2006; Breivik et al., 2009; Joyce and Pollard, 2009; Cao et al., 2010; Qiu et al., 2012, 2014; Wu et al., 2012; Fang et al., 2015; Liu et al., 2018a). Given that the external side of plasma membrane of the cellular component was important during the period of 7-14 days after TCI (see the middle columns in Figures 4B–D). We continued to examine the gene expression patterns by multiple of difference in ion channels during the development of bone cancer and BCP, on day 1, 7, and 14 after TCI treatment (Figure 6A). The statistical analysis showed that all the ion channel DEGs (*n* = 166 in each group) were altered in each comparable group starting from day 1 and remained to day 7 and 14 after TCI. Approximately 33%∼40% genes were up-regulated, in which genes with | log2 FC| ≥ 1 (10%) and | log2 FC| ≥ 2 (4%) were peaked on day 7 and declined to sham control level on day 14. In contrast, of the 166 DEGs, approximately total 60∼67% genes were down-regulated (Figure 6B). Further analysis showed that most of the up-regulated ion channel genes on day 1, 7, and 14 after TCI treatment were shared, while there were approximately 28% (15/54, day 1), 17% (10/60, day 7), and 18% (12/67, day 14) up-regulated genes were unique and seen only on the specific day after TCI treatment (Figure 6C). In consistent, most of the down-regulated genes on day 1, 7, and 14 after TCI treatment were also shared, while approximately 20% (22/112, day 1), 9.5% (10/105, day 7), and 6% (6/99, day 14) down-regulated genes were unique and seen only on the specific day after TCI treatment (Figure 6D). The up- or down-regulated top 10 genes with | log2 FC| ≥ 1, were shown by histograms in each comparable group (Figures 6E–G).

We also performed function analysis of the top 20 DEGs of ion channels in pain perception by literature retrieval and found that many have been suggested to be associated with pain perception, e.g., Ryr1(encoding ryanodine receptor, RyR1) (Ferrari et al., 2016), Cnga3 (encoding cyclic nucleotide-gated ion channel α3, CNGA3) (Heine et al., 2011), Kcnj2 (encoding inwardly rectifying potassium channel 2.1, Kir2.1) (Ma et al., 2010), Trpv4 (encoding transient receptor potential vanilloid 4, TRPV4) (Todaka et al., 2004), surprisingly, none of them to be associated with cancer pain (Figure 6H). This finding reminds us that screening genes that are related to the development of bone cancer and BCP is a necessary and urgent work in order to understand the pathogenesis of bone cancer and the associated pain.

### Divergent Gene Expression Patterns of Cytokine Signaling Following TCI Treatment

**FIGURE 7:**
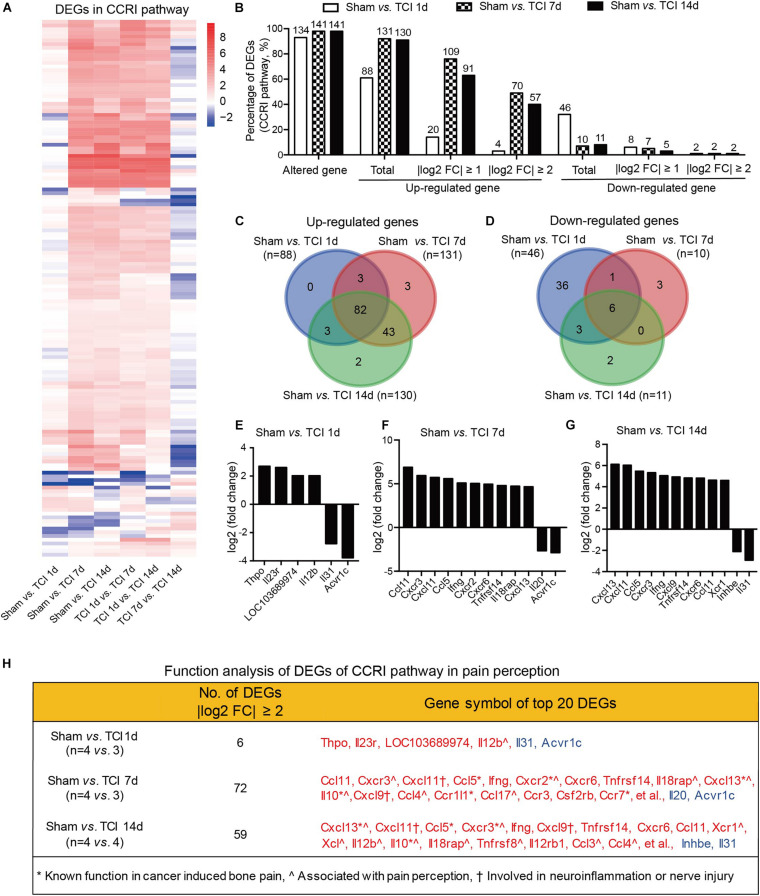
Dynamic gene expression profiles of cytokine-cytokine receptor interaction pathway during the development of BCP. **(A)** Heatmaps depicting expression patterns for genes encoding cytokine-cytokine receptor interaction pathway from pairs of comparable groups. Up- or down-regulated genes are presented as the indicated color bars (red to blue). **(B)** Histogram showing the statistics of DEGs for cytokine-cytokine receptor interaction pathway. The number and percentage of the altered gene, up- and down-regulated gene in total, with | log2 FC| ≥ 1 and | log2 FC| ≥ 2. **(C,D)** Venn diagrams present the number of up- **(C)** and down-regulated **(D)** DEGs that are unique or shared in each paired group. **(E-G)** Plot data showing the top 10 up- or down-regulated genes in cytokine-cytokine receptor interaction pathways, ranked by multiple of difference (only genes with | log2 FC| ≥ 2 were plotted). **(H)** Function analysis of top 20 DEGs in the cytokine-cytokine receptor interaction pathway that are considered associated with bone cancer pain, pain perception, as well as neuroinflammation or nerve injury. DEGs with different symbols from the encoded proteins include Acvr1c (activin A receptor type-1C), Il18rap (Interleukin 18 receptor accessory protein), Inhbe (inhibin βE subunit), Csf2rb (cytokine receptor common subunit beta).

The cytokine signaling pathways have been proved to be involved in BCP (Julius and Basbaum, 2001; Peters et al., 2005; Mantyh, 2006; Breivik et al., 2009; Joyce and Pollard, 2009; Cao et al., 2010; Qiu et al., 2012, 2014; Wu et al., 2012; Fang et al., 2015; Liu et al., 2018a). Given the ion channel gene expression patterns during the early period of development of bone cancer and BCP, we continued to show the gene expression patterns by multiple of difference in the most relevant KEGG pathway, the CCRI pathway on day 7 and 14 after TCI treatment (Figure 7A). In CCRI pathway, approximately 93-98% genes (134, 141, and 141 in different groups out of 144 total) were detected altered on day 1, 7, and 14 versus sham after TCI treatment. There were ∼61% (88/144), ∼91% (131/144), and 90% (130/144) genes up-regulated on day 1, 7, and 14, respectively, in which genes with | log2 FC| ≥ 1 (∼76%) and | log2 FC| ≥ 2 (∼63%) were peaked at day 7 and most of the up-regulation remained up till the last examination on day 14 (49 and 40%, respectively). On the other hand, the down-regulated genes were 33% (day 1), 7% (day 7), and 8% (day 14), and genes with | log2 FC| ≥ 1 were ranged from 3%∼6% and | log2 FC| ≥ 2 were rarely detected (Figure 7B). Further analysis showed that most of the up-regulated CCRI genes on day 1, 7, and 14 after TCI treatment were shared, while there were 0% (0/88, day 1), 2.29% (3/131, day 7), and 1.54% (2/130, day 14) up-regulated genes unique and seen only on the specific day after TCI treatment (Figure 7C). Such alterations of CCRI genes are greatly different from that of ion channel genes, which had more unique genes in each of the different stages after TCI treatment (see Figure 6C). As to the down-regulated CCRI genes, there were 32.6% (46 of total 141) genes down-regulated on day 1 after TCI treatment. However, only 7.1% (10/141) and 7.8% (11/141) genes were down-regulated on day 7 and 14 after TCI. There were approximately 78% (36/46, day 1) down-regulated genes were unique (Figure 7D). The top 10 genes, up- or down-regulated with | log2 FC| ≥ 2, were shown by histograms in each comparable group (Figures 7E–G).

We also performed function analysis of the top 20 DEGs from CCRI pathway in pain perception by literature retrieval and found that many have been known function in bone cancer pain or involved in neuroinflammation or nerve injury (Figure 7H). These alterations are again greatly different from the ion channel genes, in which none has been known function in bone cancer pain. These dynamic and distinct gene expression patterns demonstrate that the ion channels may be transiently involved in the very early stage (day 1-7) meanwhile cytokine signaling pathways involved in the later stages (day 7-14) of BCP development.

### Comparison of Transcriptomic Changes in Pain Models of Bone Cancer, Nerve Injury, and Inflammation

**FIGURE 8:**
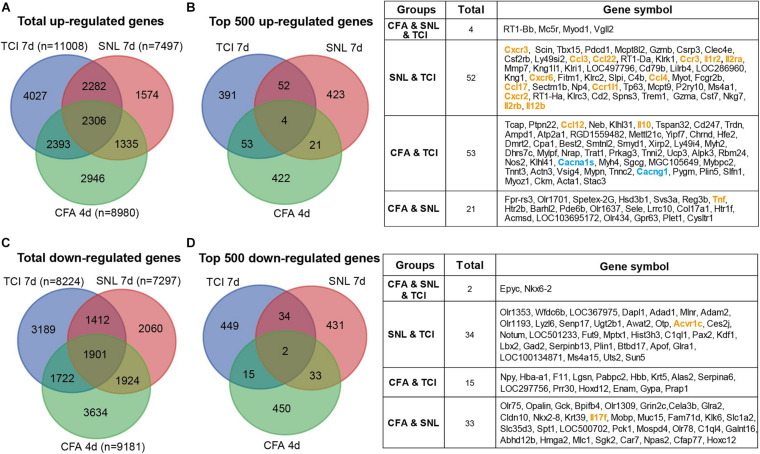
Transcriptomic comparison of the up- and down-regulated genes in rats with different forms of pain each produced by TCI, CFA, and SNL. **(A)** Venn diagrams showing numbers of the total up-regulated genes that are unique or shared by the three different pain models. **(B)** Left, Venn diagrams showing the top 500 up-regulated genes that are unique or shared by the three different pain models. Right, details of the shared genes with each comparison are listed in the table. Blue and orange fonts highlight the gene symbols for ion channels and cytokine-cytokine receptor interaction pathways, respectively. **(C)** Venn diagrams showing numbers of the total down-regulated genes that are unique or shared by the three different pain models. **(D)** Left, Venn diagrams showing the top 500 down-regulated genes that are unique or shared by the three different pain models. Right, details of the shared genes with each comparison are listed in the table. Orange fonts highlight the gene symbols for cytokine-cytokine receptor interaction pathways. Genes with different symbols from the encoded proteins include Il1r2 (interleukin-1 receptor type 2), Il2ra (interleukin-2 receptor subunit alpha), Ccr1l1 (chemokine C-C motif receptor 1-like 1), Il2rb (interleukin 2 receptor subunit beta), Cacna1s (Cav1,1), Cacng1 (voltage-dependent calcium channel gamma-1 subunit), Tnf (tumor necrosis factor).

The etiology of BCP is intricate and suggested to be composed of at least neuropathic and inflammatory factors (Falk and Dickenson, 2014). We thus continued to identify the possible common and differences in transcriptomic data from models of bone cancer pain, neuropathic pain, and acute inflammatory pain with microarray or RNAseq platform. Transcriptomic data sets [GSE129957 (Sun et al., 2020) and GSE24431 (Chang et al., 2010)] were obtained from Gene Expression Omnibus (GEO) of NCBI. We analyzed and determined proportion of up- and down regulated genes that were unique or shared among the three different pain models. Data were obtained from ipsilateral L3-L5 DRGs, L4-L6 DRGs, and L4 and L5 DRG from rats with TCI (day 7), partial sciatic nerve ligation (SNL, day 7) (Sun et al., 2020), and complete Freund’s adjuvant (CFA) injection (day 4) (Chang et al., 2010), respectively. The Venn diagrams showed that, in the total up-regulated genes from TCI (11008), SNL (7497), and CFA (8980), 20-33% genes were unique for each of the models, while there were 2306 genes (∼21-31%) shared by the three different models (Figure 8A). As shown in Figure 8B, Among the top 500 up-regulated genes in each of the three models, there were only 4 genes (0.8%, *n* = 500) shared by the three models with additional 14-20% genes shared by two of the three, while approximately 78-85% genes were unique for each of the models. The shared up-regulated genes are listed in the table (Figure 8B, right). In contrast, in the total down-regulated genes from TCI (8224), SNL (7297), and CFA (9181), there were 1901 genes (21-26%) were shared by the three different models (Figure 8C). As shown in Figure 8D, among the top 500 down-regulated genes in each of the three models, there were only 2 genes (0.4%, *n* = 500) shared in the three models with additional 10-13% genes shared by two of the three, while approximately 86-90% genes were unique for each of the models (Figure 8D). The shared down-regulated genes are listed in the table (Figure 8D, right). These findings demonstrate that, among the top 500 up- and 500 down-regulated genes, approximately 80-90% genes are unique and only 0-2% shared among the different pain models, suggesting the different pathogenesis of the pain due to bone cancer, inflammation, and nerve injury.

## Discussion

Our study reveals the transcriptomic profiles of the primary sensory neurons in the early period of bone cancer and BCP development. The major findings are four-fold: (1) TCI treatment, in addition to producing pain and bony destruction, causes alteration of thousands of genes examined in DRG that are mainly involved in the immune process, inflammatory response, and intracellular signaling transduction of carbohydrate and cytokine. The patterns of gene expression alterations were highly synchronized with the development of BCP; (2) the cAMP and calcium signaling pathways are the major processes in the initial responses to the TCI treatment including initiation of induction of BCP, while cytokine signaling and other cell-defense related pathways mainly contribute to the subsequent development of BCP, in which CCRI may play the most important roles; (3) TCI treatment-induced DEGs of ion channels may contribute to the early induction of BCP, while cytokines contribute to the maintenance of BCP. However, none of the top 20 DEGs of ion channels in pain perception in literature is associated with cancer pain, however, most of the top 20 DEGs from CCRI pathway in pain perception is involved in cancer pain; and 4) among the top 500 up- and down-regulated genes, only less than 2% genes were shared among the different forms of pain produced by bone cancer, nerve injury, and acute inflammation, and most genes were unique, indicating the difference in pathogenesis of the different forms of pain. This study reveals the uniqueness of mechanisms underlying BCP, which is, to a large extent, differently from the inflammatory and neuropathic pain and provides novel potential targets of DEGs for cancer pain.

Several lines of evidence have demonstrated cytokines and their associated signaling pathways were involved in BCP. In this study, we found that “cytokine binding”, “cytokine receptor activity”, and “cytokine activity” are highly enriched and that activation of “cytokine-cytokine receptor interaction” signal pathway exhibiting the most significant alteration after TCI treatment. Approximately 93-98% genes from the “cytokine-cytokine receptor interaction” signal pathways were altered and probably involved in the early and late stage of BCP development. Of which, cAMP and calcium signaling pathways are the major processes in the initial responses to the TCI treatment, while cytokine signaling and other cell-defense related pathways mainly contribute to the subsequent development of BCP. CCRI may play the most important roles. Among the top 20 DEGs from the “cytokine-cytokine receptor interaction” signal pathway, several genes have been reported to participate in the bone cancer pain, pain perception, neuroinflammation, or nerve injury (Garcia-Dominguez et al., 2019). The non-document DEGs, Thpo (encoding thrombopoietin, THPO), Il12rb1 (encoding interleukin-12 receptor beta 1, IL12RB1), Il23r (encoding interleukin 23 receptor, IL23R), Ccl1 (encoding chemokine C-C motif ligand 1, CCL1), Ifng (encoding cytokine interferon gamma, IFNG), Cxcr6 (encoding CXC chemokine receptor 6, CXCR6), Tnfrsf14 (encoding tumor necrosis factor receptor superfamily member 14, TNFRSF14), Ccr3 (encoding C-C chemokine receptor 3, CCR3), and Csf2rb (encoding colony stimulating factor 2 receptor beta, CSF2RB) may serve as new molecular targets for a potential therapy of BCP. Such transcriptome profiles indicate that cytokines and their associated signaling pathways may play a pivotal role in the pathogenesis of BCP, and our results provide a better understanding of the complicated inflammatory and immune processing including anti-inflammation and immunosuppression in BCP as well as the bone cancer.

Ion channels have been considered to play a pivotal role in many forms of pain including the inflammatory, neuropathic, and cancer pain. However, based on our current findings, roles of ion channels in BCP might have been over-expected. The evidence of altered DEGs of ion channels in bone cancer and BCP is weak, despite the biological process regulation of “calmodulin binding” by GO analysis and the activation of the “calcium signaling pathway” by KEGG analysis. Although all the ion channels examined here are altered in the early stage of TCI treatment, but their DEGs with | log2 FC| ≥ 2 are only 1-4%, which peaked on day 7 and quickly declined to control level within 14 days after TCI. Our function analysis of the top 20 DEGs of ion channels in pain perception by literature retrieval indicates that many ion channels have been suggested to be associated with pain perception, e.g., Ryr1 (Ferrari et al., 2016), Cnga3 (Heine et al., 2011), Kcnj2 (Ma et al., 2010), Trpv4 (Todaka et al., 2004), however, none of them to be associated with BCP (Figure 6H). This indicates that the ion channels play a very limited role in the early development of BCP and are unimportant for maintaining the established status of BCP (two weeks after TCI treatment).

By comparing the transcriptome profiles among the pain models of bone cancer, inflammation, and nerve injury, we have found that there are only small percentage of the top 500 up- or down-regulated genes shared among the pain models (0-2% by the three models and 7-11% by any two of the three models), while 78-85% genes are unique for each of the pain models. These findings speak out that the uniqueness of mechanisms underlying bone cancer and the associated BCP as well as other two forms of pain should be emphasized rather than being simply ignored, although bone cancer pain has been considered to have most characteristics of both inflammatory and neuropathic components (Falk and Dickenson, 2014).

The results in this study are supported by the previous studies on protein function. For instance, cAMP was found significantly increased in DRG on day 5 after TCI, which was synchronized with the initiation of BCP. Blocking the downstream signaling pathway of cAMP completely prevented production of BCP (Zhu et al., 2014). Furthermore, cytokines such as CCL5 (Hang et al., 2013; Pevida et al., 2014), CXCL13 (Bu et al., 2019), Il10 (Huo et al., 2018; Apryani et al., 2020) and cytokines associate receptors including CCR7 (Hirth et al., 2020), CXCR2 (Xu et al., 2014), CXCR3 (Bu et al., 2014; Hirth et al., 2020) have been reported to be involved in BCP. It is worth mentioning that CXCR3, CCR7, and IL10 have been reported to be the underlying mechanisms in cancer metastasis-induced BCP in human (An et al., 2019; Hirth et al., 2020). Especially, activation of CXCR3 has also been reported to attenuate morphine analgesia in cancer pain (Ye et al., 2014).

Rodents have been used as model animals to study human biology and diseases for decades (Breschi et al., 2017). It is reported that transcriptional patterns between orthologous organs of different species are more similar than those between different organs from the same species (Brawand et al., 2011; Barbosa-Morais et al., 2012; Merkin et al., 2012). Therefore, transcriptome data from rodents are useful to complement the study of the human diseases including neuronal disorders. However, it is also clear that with rat model alone cannot recapitulate all the features of bone cancer pain in humans, and the species differences in DEGs between rodents and humans is inevitable. As the development processes of primate nervous system are conserved across mammals (Bakken et al., 2015), future studies better employ primate model or by using human tissue slice culture to improve the usability as preclinical model for bone cancer pain research (Haehnel et al., 2019). In addition, studies have also reported the difference in pain mechanisms between males and females, thus, the findings in our current study in females need to be further investigated in males with other cancer cells probably unnecessary the female-specific breast cancer cells.

Together, this study reveals the uniqueness of mechanisms underlying BCP, which is, to a large extent, differently from the inflammatory and neuropathic pain, and provides novel potential targets of DEGs for treatment of cancer pain. Since the development of bone cancer plays an indispensable role in the induction and maintenance of bone cancer pain, future study needs to clarify the role of ion channels and cytokines in the cancer development and pain formation.

## Data Availability Statement

The datasets presented in this study can be found in the Gene Expression Omnibus (GEO) database of NCBI, and the accession number is GSE149648.

## Ethics Statement

The animal study was reviewed and approved by the Southern University of Science and Technology (SUSTech) Animal Care and Use Committee.

## Author Contributions

MZ and X-JS designed the project. MZ, SY, SL, HZ, LX, and HL conducted the experiments. MZ and X-JS analyzed data and presentation. X-JS and MZ wrote the manuscript. All authors contributed to the article and approved the submitted version.

## Conflict of Interest

The authors declare that the research was conducted in the absence of any commercial or financial relationships that could be construed as a potential conflict of interest.
